# A Comprehensive Review of Ocular Manifestations in Systemic Diseases

**DOI:** 10.7759/cureus.65693

**Published:** 2024-07-29

**Authors:** M Jayanth Kumar, Palash S Kotak, Sourya Acharya, Manikanta Nelakuditi, Avinash Parepalli

**Affiliations:** 1 Internal Medicine, Jawaharlal Nehru Medical College, Datta Meghe Institute of Higher Education and Research, Wardha, IND

**Keywords:** emerging therapies, patient management, diagnostic accuracy, interdisciplinary collaboration, systemic diseases, ocular manifestations

## Abstract

Ocular manifestations often serve as critical indicators of underlying systemic diseases, providing valuable diagnostic and prognostic information. This comprehensive review aims to elucidate the complex interplay between ocular symptoms and systemic conditions, emphasising the importance of early recognition and interdisciplinary collaboration in patient management. The review encompasses various systemic diseases, including cardiovascular, autoimmune, infectious, neurological, endocrine, hematologic, genetic, dermatologic, gastrointestinal, hepatic, renal, and connective tissue disorders, highlighting their specific ocular manifestations. Diagnostic approaches, including ophthalmologic examination techniques, imaging modalities, and laboratory tests, are discussed to enhance diagnostic accuracy. Furthermore, the review outlines current management and treatment strategies, emphasising the need for a multidisciplinary approach to care. Emerging therapies and future research directions are also explored, underscoring the necessity of continued innovation in this field. This review aims to improve clinical practices, promote integrative healthcare, and ultimately enhance patient outcomes by providing a detailed overview of ocular manifestations in systemic diseases.

## Introduction and background

The eyes, often referred to as the windows to the soul, serve as a vital indicator of systemic health [1]. Many systemic diseases present ocular symptoms that can precede or accompany other clinical signs, offering valuable insights into a patient's overall health [2]. Recognising these ocular manifestations is crucial for early diagnosis, timely intervention, and effective management of systemic diseases. The interplay between ocular and systemic health underscores the necessity for a multidisciplinary approach to patient care. Ophthalmologists, primary care physicians, and specialists must collaborate to detect and treat these manifestations, ensuring comprehensive patient management [2].

Early identification of ocular signs can lead to prompt diagnosis and treatment of potentially life-threatening systemic conditions, improving patient outcomes and quality of life. Moreover, the growing prevalence of chronic diseases such as diabetes, hypertension, and autoimmune disorders accentuates the need for increased awareness and understanding of their ocular implications [3]. With advancements in medical technology and diagnostic tools, the capacity to detect and monitor these manifestations has significantly improved, enabling more precise and targeted interventions [4].

This review aims to provide a comprehensive overview of ocular manifestations associated with various systemic diseases. The primary objectives are identifying and describing these diverse ocular symptoms and highlighting the underlying pathophysiological mechanisms. Enhancing diagnostic accuracy is crucial, involving insights into diagnostic approaches and techniques, including imaging modalities, laboratory tests, and clinical examinations. Improving interdisciplinary collaboration is also essential, emphasising the importance of communication between ophthalmologists and other healthcare providers and outlining best practices for integrated care. Additionally, the review seeks to inform clinical management and treatment strategies by discussing current and emerging therapies.

## Review

Ocular anatomy and physiology

**Figure 1:**
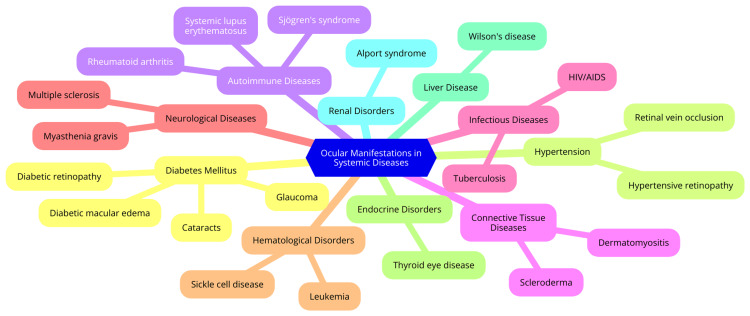
Ocular manifestations in systemic diseases Image Credit: Dr Jayant Kumar.

The eye is a highly intricate organ composed of three primary layers. The outer fibrous layer includes the sclera, the tough, protective outer layer known as the white of the eye, and the cornea, a transparent, curved front portion that refracts light. The middle vascular layer encompasses the choroid, which supplies blood to the eye, the ciliary body responsible for lens shape adjustment for focusing, and the iris, which regulates light entry. The inner neural layer features the retina, housing photoreceptor cells (rods and cones) that convert light into electrical signals, and the optic nerve, transmitting these signals to the brain [5]. Vision begins with light entering the eye. The cornea, pupil, and lens collaborate to focus light onto the retina at the eye's rear. Photoreceptor cells within the retina, including rods for low-light vision and cones for colour and high-detail vision, transform light into electrical signals. These signals travel via the optic nerve to the brain, where they are processed to form the images we perceive. The macula, a specialised region of the retina, facilitates central, detailed vision [6]. The eye is intricately linked to overall bodily health, and numerous systemic diseases can manifest ocularly. For instance, diabetes can lead to diabetic retinopathy, a major global cause of blindness. Hypertension heightens the risk of retinal vascular diseases and can exacerbate diabetic retinopathy progression. Autoimmune conditions like systemic lupus erythematosus commonly induce ocular complications such as dry eye syndrome. Additionally, systemic diseases, including acquired immunodeficiency syndrome (AIDS), rheumatoid arthritis, and Sjögren's syndrome, have been associated with various ocular effects. Ophthalmologists play a critical role in identifying and managing these ocular complications, which significantly impact patient morbidity and can indicate systemic disease activity [7]. Figure 1 illustrates ocular manifestations in systemic diseases.

Cardiovascular diseases

Hypertension significantly increases the risk of developing retinal vascular diseases, notably hypertensive retinopathy. This condition arises from damage to retinal blood vessels due to sustained high blood pressure. Symptoms may include double vision, blurred vision, and headaches. If untreated, hypertensive retinopathy can progress to severe complications such as retinal vein or artery occlusion, ischemic optic neuropathy, and malignant hypertension. Timely detection and management of hypertension are critical in preventing vision loss [8]. Diabetic retinopathy stands as the most prevalent microvascular complication of diabetes and a leading cause of blindness worldwide. Almost all individuals with type 1 diabetes and about 60% of those with type 2 diabetes develop some form of retinopathy within two decades, regardless of their diabetes management. Diabetic macular oedema, characterised by fluid accumulation in the retina, is a primary cause of vision impairment in diabetic retinopathy. Effective treatments, including laser therapy and intravitreal injections, can manage the condition if administered promptly [9].

Atherosclerosis, the accumulation of fatty deposits in arteries, affects ocular vasculature and can lead to conditions such as retinal artery or vein occlusion, central retinal artery occlusion, and embolic events. These conditions often result in sudden vision loss and necessitate swift diagnosis and management. Atherosclerosis also heightens the risk of developing diabetic retinopathy and other ocular complications [10]. Various cardiovascular diseases can manifest with ocular symptoms. For instance, carotid and vertebral artery diseases can cause amaurosis fugax, a transient vision loss. Infective endocarditis may lead to Roth spots, retinal emboli, and sub-retinal abscesses. Uncommon cardiac conditions like myxomas can provoke central retinal artery occlusion and sudden vision loss. Ophthalmologists are crucial in identifying and treating these ocular manifestations, impacting patient well-being and indicating underlying systemic disease activity. Early recognition and appropriate management are essential to preserve vision and monitor cardiovascular health effectively [11].

Autoimmune and inflammatory disorders

Rheumatoid arthritis (RA), a systemic autoimmune disorder, often manifests with significant ocular complications. One of the most prevalent among RA patients is dry eye syndrome, also known as keratoconjunctivitis sicca, affecting up to 30% of individuals. This condition stems from dysfunction of the lacrimal and meibomian glands, leading to symptoms like a gritty sensation, burning, reduced visual clarity, and sensitivity to light. Episcleritis is another common ocular manifestation in RA, impacting as many as 66% of patients. It involves inflammation of the episclera, presenting with redness and sometimes associated with systemic symptoms such as fatigue and joint pain [12]. Additionally, scleritis, characterised by the inflammation of the sclera (the eye's white outer layer), occurs in about 23% of RA cases and poses a risk of severe complications like corneal perforation or globe rupture [13]. Less frequently, RA may lead to peripheral ulcerative keratitis, uveitis, retinal vasculitis, and glaucoma, all of which can threaten vision if not promptly managed [14]. Given the potential severity of these conditions, ophthalmologists play a crucial role in the comprehensive care of RA patients. Regular eye examinations are essential for early detection and effective management, aiming to mitigate vision loss and optimize patient outcomes [15].

Infectious diseases

Human immunodeficiency virus (HIV) and acquired immunodeficiency syndrome (AIDS) can lead to various ocular conditions due to compromised immune function [16]. The most common ocular complication in HIV/AIDS patients is cytomegalovirus (CMV) retinitis, an opportunistic infection that can cause retinal detachment and severe vision loss if untreated. Cotton wool spots, characterised by fluffy white spots on the retina, typically indicate immune system compromise. Kaposi's sarcoma, a vascular tumour that may appear on the eyelid or sclera, can cause significant cosmetic concerns and, in some cases, vision impairment. Dry eye syndrome (keratoconjunctivitis sicca) affects approximately 10%-20% of HIV-positive individuals, often in advanced disease stages [17]. Syphilis can manifest in the eye with conditions like conjunctivitis, uveitis, and optic neuritis. In HIV-positive patients, syphilis-related ocular complications can be more severe, including interstitial keratitis, iritis, and choroiditis, potentially leading to significant visual impairment and blindness if untreated [18]. Tuberculosis can cause ocular manifestations, such as anterior and posterior uveitis and choroiditis. In HIV-positive patients, tuberculosis-related eye issues may be more severe due to immune compromise. Lyme disease can also affect the eye, causing conditions like conjunctivitis, keratitis, and uveitis, with the potential for inflammation and vision loss as the infection spreads via the bloodstream [19]. Herpes simplex virus (HSV) can lead to keratitis, uveitis, and retinitis. In HIV-positive individuals, HSV infections may be more severe and recurrent due to immune suppression. Herpes zoster ophthalmicus (HZO), caused by the varicella-zoster virus (VZV), can cause severe eye pain, conjunctivitis, keratitis, and uveitis, potentially resulting in significant vision impairment [20]. Treatment options include antiviral medications for HSV and HZO, antibiotics for bacterial infections such as syphilis and tuberculosis, antifungal drugs for fungal infections like candida, and specific antiviral therapies like ganciclovir and foscarnet for CMV retinitis. Early detection and appropriate treatment are crucial to prevent vision loss and manage ocular complications associated with these infectious diseases [21].

Neurological disorders

Optic neuritis is a common manifestation of multiple sclerosis (MS), affecting up to 50% of patients. Often, the first sign of MS presents as acute, painful vision loss in one eye, typically improving within a month. Patients with optic neuritis and MRI findings consistent with MS lesions have a heightened risk of developing clinically definite MS within a few years [22]. Myasthenia gravis, another autoimmune disorder, can impact ocular muscles, leading to symptoms like ptosis (drooping eyelid) and diplopia (double vision). These ocular signs often precede generalised muscle weakness and may fluctuate, worsening with activity and improving with rest, which is a characteristic of the disease [23]. Parkinson's disease and Alzheimer's disease, both neurodegenerative disorders, can also exhibit ocular manifestations. Parkinson's patients may experience dry eyes, double vision, and impaired pupillary reflexes due to underlying neurodegeneration and autonomic dysfunction. Alzheimer's patients often present with poor visual acuity, difficulty interpreting visual information, and pupillary abnormalities, significantly affecting daily functioning [24]. Strokes affecting visual pathways can cause various visual field defects, such as homonymous hemianopia, where half of the visual field is lost in both eyes. Recognising these defects promptly aids in diagnosing stroke location and guiding patient rehabilitation [25]. Neurological disorders can lead to a broad spectrum of ocular effects, from vision loss and pupillary abnormalities to eye movement disorders and dry eyes. Ophthalmologists are crucial in identifying and managing these neuro-ophthalmic complications, impacting patient quality of life and serving as vital diagnostic indicators for underlying neurological conditions [26].

Endocrine disorders

Thyroid eye disease, or Graves ophthalmopathy, is an autoimmune condition where the immune system targets tissues around the eyes. This results in symptoms such as redness, pain, swelling around the eyes, bulging of the eyes (proptosis or exophthalmos), restricted eye movements, double vision, and, in severe cases, corneal exposure, leading to potential vision loss. These ocular manifestations often precede the onset of thyroid dysfunction and are typically the initial signs of Graves' disease. Treatment focuses on managing inflammation and preventing vision impairment [27]. Pituitary adenomas can cause various ocular complications depending on their size and location. Larger tumours may compress the optic chiasm, resulting in visual field defects like bitemporal hemianopia. Ophthalmoplegia is another common finding due to compression of the cavernous sinus and cranial nerves III, IV, and VI. Tumours extending into the orbit can lead to proptosis and compression of the optic nerve. Early detection and treatment, often through surgical intervention, are crucial in preserving vision and maintaining eye function in patients with pituitary tumours [28]. Other endocrine disorders also exhibit ocular manifestations. Cushing's syndrome can cause periorbital fat deposition, eyelid retraction, and exophthalmos. Acromegaly from excess growth hormone may lead to proptosis and optic nerve compression. Hypothyroidism is associated with eyelid oedema, periorbital myxoedema, and optic neuropathy. In summary, the eyes serve as critical indicators of underlying endocrine disorders, highlighting the essential role of ophthalmologists in the comprehensive management of these complex conditions [29].

Hematologic and oncologic disorders

Leukemic retinopathy is leukaemia's most common ocular complication, affecting up to 90% of patients. It primarily results from haematological abnormalities such as anaemia and thrombocytopenia, leading to retinal haemorrhages, cotton wool spots, and Roth spots [30]. In addition to these indirect effects, leukaemia can directly infiltrate the retina, optic nerve, and iris. Optic nerve head swelling due to increased intracranial pressure, cranial nerve palsies, opportunistic infections, and chemotherapy-related issues like cataracts and ocular hypertension are other ocular manifestations associated with leukaemia [31]. Leukemic retinopathy is more prevalent in acute forms of leukaemia compared to chronic types, and ocular involvement may precede systemic disease, occur during relapse, or develop during remission phases. In lymphoma patients, ocular involvement occurs in 5%-30% of cases. Common manifestations include direct infiltration of the orbit, conjunctiva, uvea, lacrimal gland, compressive mass lesions, corneal deposits, and hyperviscosity retinopathy. Vision loss can occur from optic nerve infiltration or exudative retinal detachment, either unilaterally or bilaterally [32]. Anaemia associated with leukaemia and lymphoma contributes to retinal changes like haemorrhages, cotton wool spots, and optic nerve pallor, with the severity correlating with the degree of anaemia. High-dose glucocorticoids used in leukaemia and lymphoma treatment can lead to ocular hypertension, cataracts, diplopia, and other ocular side effects. Total body irradiation is also linked to various ocular complications [33].

Genetic and metabolic disorders

Marfan syndrome, an inherited connective tissue disorder, can affect several eye parts, including the cornea, lens, retina, and optic nerve. The most common ocular manifestation is ectopia lentis (lens subluxation), observed in 60%-87% of patients and often as an initial sign of the disease. Other ocular features include myopia, astigmatism, retinal detachment, glaucoma, and strabismus. Regular ophthalmological exams are essential for early detection and management of these ocular complications to prevent vision loss [34]. Wilson's disease, a copper metabolism disorder, produces copper deposition in various organs, including the eyes. Ocular manifestations include Kayser-Fleischer rings (copper deposits in the cornea), sunflower cataracts, and optic neuropathy. These eye findings can provide crucial diagnostic clues, particularly in asymptomatic patients [35]. Mitochondrial disorders encompass a group of genetic conditions affecting mitochondrial function, presenting diverse ocular manifestations such as optic neuropathy, retinal degeneration, external ophthalmoplegia, ptosis, and corneal endothelial dystrophy. Early recognition of these ocular signs is vital for diagnosing and managing these complex multisystem disorders [36]. Numerous inherited metabolic disorders can involve the eyes, leading to conditions like corneal opacities, cataracts, retinal degeneration, optic nerve abnormalities, and disorders of the extraocular muscles. Examples include mucopolysaccharidoses, lipid storage disorders, and amino acid disorders. Ophthalmological assessment is critical in diagnosing these conditions, monitoring disease progression, and guiding treatment strategies [37].

Dermatologic conditions

Psoriasis, a chronic inflammatory skin condition, can extend its effects to the eyes and periocular area, leading to various ocular manifestations. The most common complication is keratoconjunctivitis sicca (dry eye syndrome), affecting up to 50% of psoriasis patients. Blepharitis, inflammation of the eyelids, is also prevalent, occurring in up to 47% of patients with moderate-to-severe psoriasis [38]. Conjunctivitis, characterised by conjunctival redness, is another frequent issue among psoriasis patients. Uveitis, inflammation of the uvea, is more commonly observed in individuals with psoriatic arthritis, while keratitis and cornea inflammation can also occur [39]. Less commonly, psoriasis can lead to complications such as cataracts, retinal pathologies, entropion (inward eyelid turning), and ectropion (outward eyelid turning). These ocular manifestations may result from direct involvement of the eye by psoriatic plaques, immune-mediated inflammatory processes, or adverse effects of psoriasis treatments [38]. Screening for ocular involvement is crucial to enable early detection and management of these complications, particularly in patients with moderate-to-severe psoriasis. Prompt recognition and treatment are essential to prevent vision-threatening issues and enhance the overall quality of life for individuals affected by psoriasis [40].

Gastrointestinal and hepatic diseases

Inflammatory bowel disease (IBD), encompassing conditions like Crohn's disease and ulcerative colitis, can present with significant ocular manifestations affecting up to 10% of patients. Crohn's disease tends to have a higher prevalence of eye-related complications than ulcerative colitis. The most common ocular issues associated with IBD include uveitis, episcleritis, scleritis, and dry eye syndrome [41]. Uveitis, which can affect both the anterior and posterior segments of the eye, often correlates with joint and skin symptoms of IBD and may precede the diagnosis of gastrointestinal involvement. Episcleritis and scleritis, characterised by inflammation of the episclera and sclera, respectively, can occur during active phases of intestinal disease. Additionally, dry eye syndrome, or keratoconjunctivitis sicca, is a frequent complication observed in IBD patients [42]. Ocular symptoms in IBD can be nonspecific, making early detection and diagnosis challenging. Therefore, maintaining a high index of suspicion and conducting regular ophthalmologic evaluations are crucial for patients with IBD to ensure timely detection and management of these ocular manifestations [43]. Hepatic diseases, such as primary biliary cholangitis (PBC) and general liver disease, can also lead to ocular complications. PBC may manifest with xanthelasma (yellow lid lesions due to hyperlipidaemia and prolonged cholestasis) and dry eye syndrome. Liver disease, in general, is associated with cataract development, often due to nutritional deficiencies related to the underlying condition, as well as dry eye syndrome [44]. Malabsorption syndromes like celiac disease can result in ocular complications, primarily dry eye syndrome. Recognising these ocular manifestations associated with gastrointestinal and hepatic diseases is crucial, as early detection and management can help prevent vision loss and guide the treatment of the underlying systemic condition [45].

Renal diseases

Patients with chronic kidney disease (CKD) face an elevated risk of developing various ocular complications that can significantly impact vision. One of the most significant risks is retinopathy, including diabetic retinopathy, hypertensive retinopathy, and other retinal microvascular changes. These retinal microvascular changes can predict CKD development, underscoring the close connection between kidney and eye health [46]. Glaucoma is another prevalent ocular condition among CKD patients, likely attributable to shared risk factors such as diabetes and hypertension. Close monitoring is essential to detect and manage glaucoma promptly, as untreated cases can lead to vision loss [47]. Cataracts are common in CKD patients, who are at a heightened risk compared to the general population. This increased risk may stem from metabolic disturbances and oxidative stress associated with chronic renal failure [48]. Beyond these well-established ocular complications, chronic renal failure can lead to more severe and vision-threatening conditions like vitreous haemorrhage, retinal detachment, neovascular glaucoma, and accelerated cataract formation. Certain medications used in CKD management, such as diuretics and calcium channel blockers, have also been linked to the progression of glaucoma [46]. Even CKD patients undergoing haemodialysis are susceptible to ocular complications. Treatments like interferon therapy for renal cell carcinoma, for instance, can induce retinopathy characterised by cotton wool spots and retinal haemorrhages [49]. Vigilant screening and timely intervention are crucial to preserve vision and manage these ocular challenges effectively in CKD patients.

Connective tissue disorders

Marfan syndrome is an autosomal dominant connective tissue disorder known for its ocular, cardiovascular, and skeletal abnormalities. One of the most common ocular manifestations is ectopia lentis, where the lens is dislocated or subluxated, occurring in 60%-87% of patients. This condition can lead to visual impairment due to irregular astigmatism and glare. Other ocular features of Marfan syndrome include myopia, astigmatism, keratoconus, glaucoma, retinal detachment, and strabismus. Retinal detachment, occurring in 5%-26.5% of cases and often affecting both eyes, is a significant concern [50]. Ehlers-Danlos syndrome (EDS) comprises a group of inherited connective tissue disorders characterised by joint hypermobility, skin hyperextensibility, and tissue fragility. Ocular manifestations in EDS include keratoconus, keratoglobus, high myopia, retinal detachment, and strabismus. Keratoconus, a progressive thinning and irregular cornea curvature, is particularly prevalent, affecting up to 30% of patients. Retinal detachment is also a serious complication, with reported incidences ranging from 1% to 5% [51]. Scleroderma, or systemic sclerosis, is an autoimmune connective tissue disorder known for its skin and internal organs fibrosis. Ocular manifestations in scleroderma include eyelid changes (such as telangiectasia, oedema, and fibrosis), dry eye syndrome, corneal epithelial changes, scleritis, and retinal vascular occlusions. Fibrosis of the eyelids and periorbital tissues can lead to conditions like lagophthalmos, exposure keratopathy, and corneal ulceration. Scleroderma patients also face an increased risk of developing glaucoma due to angle closure resulting from the fibrotic process [52].

Paediatric considerations

Congenital disorders can present a diverse array of ocular manifestations in paediatric patients. Down syndrome, for instance, is associated with conditions like blepharitis, strabismus, nystagmus, refractive errors, cataracts, and glaucoma. These ocular issues often require early detection and management to mitigate potential vision impairment [53]. Marfan syndrome can manifest in paediatric patients with ectopia lentis (lens dislocation), myopia, and a heightened risk of retinal detachment, emphasising the need for regular ophthalmological monitoring [53]. Albinism commonly presents with nystagmus, photophobia, strabismus, and foveal hypoplasia, necessitating specialised care to address visual challenges from early childhood [53]. Metabolic disorders such as cystinosis and mucopolysaccharidosis can lead to corneal changes and optic nerve damage, highlighting the importance of comprehensive ophthalmologic evaluation in affected children [53]. Paediatric autoimmune disorders frequently involve the eyes. Juvenile idiopathic arthritis is a significant cause of uveitis in children. It can also lead to conditions like band keratopathy and cataracts, underscoring the need for timely intervention to prevent complications [54]. Systemic lupus erythematosus may present with keratoconjunctivitis sicca, discoid lupus lesions on the eyelids, and potentially vision-threatening retinopathy, necessitating close collaboration between ophthalmologists and rheumatologists [54]. Infectious diseases in children can also have profound ocular implications. Congenital toxoplasmosis, for instance, may result in chorioretinitis and optic neuritis. In contrast, congenital rubella infection can cause cataracts, glaucoma, and retinopathy, highlighting the critical role of ophthalmologists in early diagnosis and management [55]. Given that ocular findings often serve as initial indicators of underlying systemic conditions in paediatric patients, early recognition and comprehensive management of these manifestations are crucial in preserving vision and guiding effective treatment strategies. Ophthalmologists play a pivotal role in the multidisciplinary care of these complex paediatric cases [56].

Diagnostic approaches

Ophthalmologic examination techniques encompass a comprehensive array of tests conducted by ophthalmologists or optometrists to evaluate vision and ocular health. These assessments include evaluating visual acuity, testing visual fields through confrontation, examining pupillary responses, assessing eye movements and alignment, testing binocular vision, utilising the Amsler grid, conducting slit-lamp biomicroscopy, performing indirect ophthalmoscopy, measuring intraocular pressure (tonometry), and evaluating the angle of the anterior chamber (gonioscopy). These tests yield valuable insights into the eye's structural integrity and functional capabilities [57]. Imaging modalities play a pivotal role in diagnosing and managing various ocular conditions. Optical coherence tomography (OCT) offers high-resolution images of the retina and optic nerve, facilitating the detection of subtle changes. Magnetic resonance imaging (MRI) provides detailed images of the orbit and adjacent structures, while computed tomography (CT) produces cross-sectional images of these areas. These advanced imaging techniques empower ophthalmologists to diagnose more precisely and formulate tailored treatment strategies [58]. Laboratory tests also support the diagnosis and management of diverse eye disorders. Polymerase chain reaction (PCR) can detect genetic mutations or viral infections, while flow cytometry analyzes cellular characteristics within the eye. Additional laboratory methods, such as analysing blood, tissue, and other biological fluids, furnish valuable diagnostic insights [59]. Genetic testing is critical in diagnosing and managing inherited eye conditions like retinitis pigmentosa and other forms of genetic retinal degeneration. By identifying genetic mutations, ophthalmologists can better comprehend the underlying mechanisms and devise targeted treatment plans. Genetic testing also aids in managing systemic diseases that manifest ocular symptoms [60].

Management and treatment strategies

A collaborative, multidisciplinary team approach involving ophthalmologists, rheumatologists, immunologists, and other specialists is essential for effectively managing ocular complications associated with systemic diseases. This integrated approach allows for thorough assessment, personalised treatment plans, and coordinated follow-up care to address the wide spectrum of ocular manifestations that can arise [61]. Treatment strategies are tailored based on the specific ocular involvement. For severe cases such as retinopathy in systemic lupus erythematosus, options may include topical therapies or systemic immunosuppression. Conditions like diabetic retinopathy and macular oedema may require a combination of treatments such as laser photocoagulation, intravitreal anti-vascular endothelial growth factor (VEGF) injections, and corticosteroid implants to manage complications that threaten vision. Effective management of the underlying systemic disease, such as rigorous blood pressure control for hypertension, is crucial for preventing and mitigating ocular complications [62]. Ophthalmologists play a pivotal role in educating patients about the ocular manifestations associated with their systemic condition and stressing the importance of regular eye examinations for early detection. Coordinated follow-up care between ophthalmologists and other specialists ensures timely monitoring and management of ocular complications, essential for preserving vision and guiding the treatment of systemic disease [63].

Future directions

Recent advances in research and emerging therapies offer significant promise for enhancing the management of ocular manifestations associated with systemic diseases. Early detection of ocular changes is critical, as they can precede or coincide with the progression of systemic diseases. Innovations in imaging technologies such as optical coherence tomography (OCT) and widefield retinal imaging enable more comprehensive monitoring of ocular health, facilitating earlier intervention [64]. Ongoing research is exploring targeted immunotherapies for autoimmune disorders like systemic lupus erythematosus (SLE) and thyroid eye disease. These novel treatments aim to modulate specific immune pathways in developing ocular manifestations, presenting more effective and personalised therapeutic options. By addressing underlying immune dysregulation, these therapies hold the potential to better manage ocular complications associated with systemic diseases [65]. In severe cases of ocular surface diseases like Stevens-Johnson syndrome, regenerative approaches are showing promise. Advanced therapies utilising techniques such as amniotic membrane transplantation and cultivated cell grafts have demonstrated the ability to restore the ocular surface and prevent vision loss. These regenerative strategies represent a promising frontier in managing debilitating ocular complications arising from systemic conditions [66]. Effective management of systemic diseases with ocular involvement necessitates close collaboration among ophthalmologists, primary care physicians, and relevant specialists. Integrating ocular assessments into routine disease monitoring can facilitate early intervention and preserve vision. This multidisciplinary approach is crucial for understanding the patient's overall health and the complex interplay between systemic conditions and their ocular manifestations [67]. Lastly, improving patient education on the ocular risks associated with systemic diseases and the importance of regular eye examinations is paramount. Empowering patients to actively monitor their eye health and seek timely ophthalmic care can lead to earlier detection and more effective management of ocular complications. Increased patient awareness and engagement can significantly contribute to preserving vision and enhancing outcomes for individuals with systemic conditions affecting the eyes [68].

## Conclusions

The intricate relationship between ocular manifestations and systemic diseases underscores the critical role of the eye in reflecting overall health. This comprehensive review has highlighted the importance of understanding these manifestations for early diagnosis, accurate management, and effective treatment of systemic conditions. By identifying and describing the various ocular signs associated with systemic diseases, enhancing diagnostic accuracy, promoting interdisciplinary collaboration, and informing clinical management strategies, this review aims to improve patient care outcomes. The advancements in diagnostic tools and treatment modalities have significantly enhanced our ability to promptly detect and address these ocular manifestations. However, continued research and innovation are essential to bridge existing knowledge gaps and explore new frontiers in understanding the ocular implications of systemic diseases. Fostering an integrative approach to healthcare, where ophthalmologists and other healthcare providers work collaboratively, will lead to better health outcomes and a higher quality of life for patients.
